# Can large language models predict antimicrobial peptide activity and toxicity?†

**DOI:** 10.1039/d4md00159a

**Published:** 2024-04-23

**Authors:** Markus Orsi, Jean-Louis Reymond

**Affiliations:** a Department of Chemistry, Biochemistry and Pharmaceutical Sciences, University of Bern Freiestrasse 3 3012 Bern Switzerland jean-louis.reymond@unibe.ch

## Abstract

Antimicrobial peptides (AMPs) are naturally occurring or designed peptides up to a few tens of amino acids which may help address the antimicrobial resistance crisis. However, their clinical development is limited by toxicity to human cells, a parameter which is very difficult to control. Given the similarity between peptide sequences and words, large language models (LLMs) might be able to predict AMP activity and toxicity. To test this hypothesis, we fine-tuned LLMs using data from the Database of Antimicrobial Activity and Structure of Peptides (DBAASP). GPT-3 performed well but not reproducibly for activity prediction and hemolysis, taken as a proxy for toxicity. The later GPT-3.5 performed more poorly and was surpassed by recurrent neural networks (RNN) trained on sequence-activity data or support vector machines (SVM) trained on MAP4C molecular fingerprint-activity data. These simpler models are therefore recommended, although the rapid evolution of LLMs warrants future re-evaluation of their prediction abilities.

## Abstract 2

The large language models GPT-3 and GTP-3.5 were challenged to predict the activity and hemolysis of antimicrobial peptides from their sequence and compared to recurrent neural networks and support vector machines.

## Graphical abstract

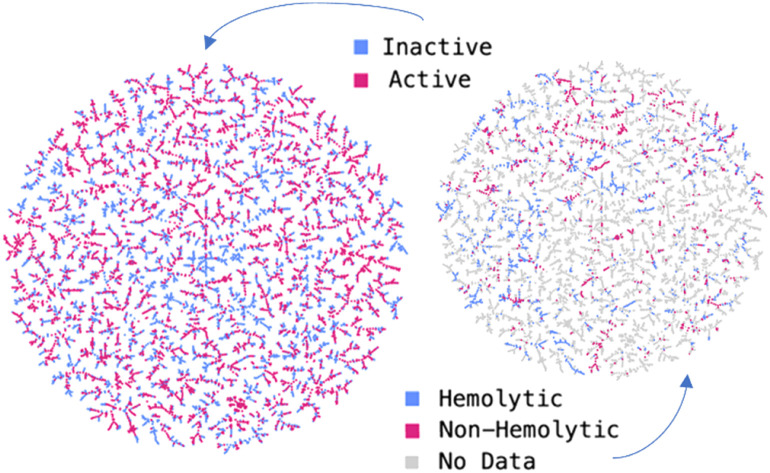

## Introduction

Antimicrobial peptides (AMPs) have gained significant attention in the field of drug discovery due to their potential therapeutic applications in the fight against antimicrobial resistance.^1–3^ However, the vast number of possible peptide sequences and their complex structure–activity relationship landscape mean that it is difficult to rationally design peptides with the desired biological activity, in particular tuning their activity *versus* toxicity to human cells, which is often measured as hemolysis of human red blood cells.^4,5^

To address this issue, several machine-learning models have been developed for the *de novo* design of antimicrobial peptides.^6–21^ Because property prediction from a peptide sequence can be framed as a natural language processing problem, many of these models use architectures specifically designed for language processing tasks.^22–24^ Furthermore, the emergence of large language models (LLMs), such as OpenAI's GPT models,^25^ has opened new possibilities for leveraging powerful language processing capabilities in drug discovery applications. Recent attempts by Jablonka *et al.* to explore the capabilities of GPT-3 for predicting properties of small molecules in various applications have shown that GPT-3 was able to perform comparably or even outperform conventional statistical models, particularly in the low data regime.^26^ There also have been successful efforts into augmenting LLM capabilities to tackle tasks related to small molecule chemistry in the areas of organic synthesis, drug discovery, and materials design.^27–30^ Hereby, the models mainly orchestrate a set of tools to solve chemistry tasks starting from a natural language prompt.^31–33^ However, to the best of our knowledge LLMs have not been implemented to predict the bioactivity of peptides yet.

In this study, we aimed to compare GPT models fine-tuned on antimicrobial peptide sequence data with models that have been previously used to predict antimicrobial activity and hemolysis of peptide sequences.^13,14^ Alongside evaluating the performance of the fine-tuned GPT models, we also seek to explore the advantages and disadvantages they offer in terms of time and cost effectiveness. Furthermore, we compare the performance of models trained on amino acid sequences to a support-vector machine (SVM) trained on the MAP4C fingerprint.^34^

## Methods

### Datasets

The datasets used in this study were peptide sequences with annotated antimicrobial and hemolytic activity collected from the Database of Antimicrobial Activity and Structure of Peptides (DBAASP).^13,35^ Sequences exhibiting an activity measure below 10 mM, equivalent to 10 000 nM or 32 mg mL^−1^, against at least one of the selected target organisms *P. aeruginosa*, *A. baumannii*, or *S. aureus* were categorized as active. Conversely, sequences with activity measures exceeding 10 mM, 10 000 nM, or 32 mg mL^−1^ against all of these targets were categorized as inactive. When available, activity against human erythrocytes was utilized to classify sequences as either hemolytic or non-hemolytic. Concentrations were standardized to mM, and sequences causing less than 20% hemolysis at concentrations equal to or above 50 mM were categorized as non-hemolytic and flagged accordingly. Sequences inducing more than 20% hemolysis were classified as hemolytic, irrespective of concentration. The dataset used for the classification tasks contained 9548 (7160 training/2388 validation) sequences with annotated antimicrobial activity, of which 2262 (1723 training/539 validation) sequences had additional hemolytic activity annotations. To test models in low data regimes, we randomly selected subsets from the original training sets, representing approximately 20% and 2% of the original activity set, and approximately 10% of the original hemolysis set. All datasets are further described in Table 1. To ensure consistency, we maintained the same training and test split for all initial evaluations. For the detailed study, we used the same 5-fold cross-validation sets.

**Table 1:** Sizes and composition of the datasets used in the present study. Datasets are available at https://github.com/reymond-group/LLM_classifier

Name	Size	# positive class	# negative class
Activity training	7160	3580	3580
Activity training 20%	1400	701	699
Activity training 2%	140	74	66
Activity validation	2388	1194	1194
Hemolysis training	1723	717	1006
Hemolysis training 10%	170	65	105
Hemolysis validation	539	226	313

### Models

As reference models, we used our previously reported naïve Bayes (NB), support vector machine (SVM), random forest (RF), and recurrent neural network (RNN) classifiers trained on the same data.^13^ We furthermore trained two additional SVM models on alternative representations of peptide sequences: one utilizing the MAP4C fingerprint^34^ with a custom Jaccard kernel, and another using predicted fraction of helical residues and hydrophobic moment with a linear kernel. Fraction of helical residues were predicted using SPIDER3.^36^ Hydrophobic moment was computed using the method of Eisenberg *et al.*^37^

To explore the potential of GPT-3 models for antimicrobial and hemolytic activity classification, we performed fine-tuning of the Ada, Babbage, and Curie models which were accessible through the OpenAI API (v0.28.0, accessed between 25.05.2023 and 01.06.2023). The fine-tuning process involved training each model using the full, 20% and 2% sets for activity classification and the full and 10% set for the hemolysis classification. In the later evaluation with the more advanced LLM GPT-3.5 Turbo, fine-tuning was also performed *via* OpenAI's Python API (v1.11.1), following the provided guidelines, but we restricted ourselves to the full model. The utilized fine-tuning datasets contained a system role (“predicting antimicrobial activity/hemolysis from an amino acid sequence”), a user message (peptide sequence formatted as “SEQUENCE ->”), and a system message (“0” for negative labels and “1” for positive labels).

### Metrics

All models were evaluated using five commonly accepted performance metrics: ROC AUC, accuracy, precision, recall and F1. Metrics were either calculated using the scikit-learn (v1.4.0) Python (v3.12.1) package (reference models and GPT-3.5) or directly obtained from the OpenAI platform after fine-tuning was completed (for all GPT-3 models).

#### ROC AUC (receiver operating characteristic area under the curve)

The ROC AUC measures the area under the receiver operating characteristic curve, which plots the true positive rate (sensitivity) against the false positive rate. A higher ROC AUC value (ranging from 0 to 1) indicates better discrimination and predictive performance of the model.

#### Accuracy

Accuracy measures the overall correctness of the model's predictions, calculating the ratio of correctly classified instances to the total number of instances. It provides a general understanding of the model's performance but can be misleading in imbalanced datasets.
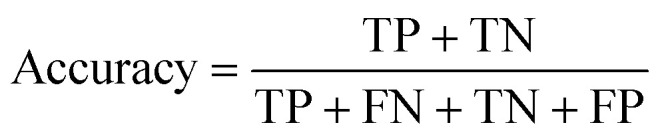


#### Precision

Precision measures the proportion of true positives out of all predicted positives. It focuses on the model's ability to avoid false positives.
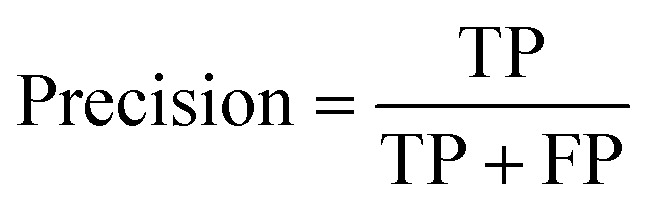


#### Recall

Recall measures the proportion of true positives out of all actual positives. It represents the model's ability to identify positive instances accurately.
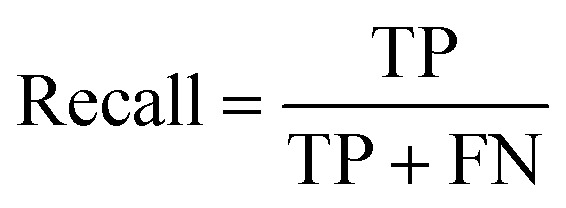


#### F1 score

F1 is the harmonic mean of precision and recall. It provides a balanced measure that considers both precision and recall.
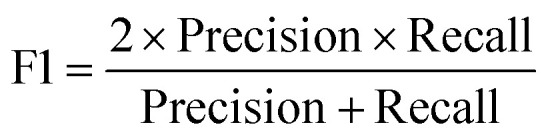


## Results and discussion

### Model screening

Starting from the DBAASP dataset of 9548 peptide sequences annotated with antibacterial activity and 2262 peptide sequences annotated with hemolysis effect, we had previously evaluated NB, RF, SVM and RNN models, and found the latter to perform best for predicting both activity and hemolysis from sequence data.^13,14^ For additional reference, we trained an SVM on the fraction of helical residues and the hydrophobic moment, two properties commonly known to correlate with antimicrobial activity, as well as another SVM on MAP4C, a molecular fingerprint that can reliably encode large molecules such as natural products and peptides including their chirality,^34^ a parameter which we considered important since our data listed sequences containing both l- and d-amino acids.

Aiming to test how LLMs perform in predicting antimicrobial activity and hemolysis, we first fine-tuned and evaluated GPT-3 Ada, Babbage, and Curie models. As discussed in our preprint, these models performed slightly better than the reference models, and even provided good performances when trained in low data regime (20% and 2% of full data). However, these models were later deprecated by OpenAI and their performance cannot be reproduced. We therefore discuss herein only the results obtained with the more recent GPT-3.5 model, in comparison with the reference models.

For both, prediction of antimicrobial activity and prediction of hemolysis, the top-performing models were the MAP4C SVM and the RNN model trained on sequence data, the latter being the best performer in our original work (Table 2).^13^ The performances for both models were in a similar range, although the RNN displayed a notably higher ROC-AUC in both tasks. GPT-3.5 displayed the highest recall performance among the activity models, indicative of the model's tendency to overly favor positive predictions, potentially leading to increased false positive predictions. On the other hand, the features SVM trained only on helicity and hydrophobic moment did not perform significantly above background, and was later used as a negative control model.

**Table 2:** Performance metrics of all models tested on antimicrobial activity and hemolysis classification. The best value for each metric is highlighted in bold. NB: naïve Bayes, RF: random forest, SVM: support vector machine, RNN: recurrent neural network, MAP4C: chiral MinHashed atom-pair fingerprint of diameter 4, GPT: generative pre-trained transformer

Model	ROC AUC	Accuracy	Precision	Recall	F1
NB act.	0.55	0.55	0.59	0.32	0.42
RF act.	0.81	0.71	0.7	0.75	0.73
SVM act.	0.75	0.68	0.68	0.68	0.68
RNN act.	**0.84**	0.76	0.74	0.8	0.77
Features SVM act.	0.65	0.65	0.66	0.62	0.64
MAP4C SVM act.	0.8	**0.8**	**0.79**	0.83	**0.8**
GPT-3.5 Turbo act.	0.68	0.68	0.62	**0.93**	0.75
NB hem.	0.58	0.56	0.48	0.76	0.59
RF hem.	0.8	0.77	**0.81**	0.6	0.69
SVM hem.	0.69	0.73	0.72	0.58	0.65
RNN hem.	**0.87**	0.76	0.7	0.76	0.73
Features SVM hem.	0.62	0.63	0.57	0.5	0.54
MAP4C SVM hem.	0.83	**0.83**	0.76	**0.85**	**0.8**
GPT-3.5 Turbo hem.	0.65	0.69	0.72	0.43	0.54

### Model comparison

Following the initial model screening, we aimed to validate our findings through a more robust approach: a 5-fold cross-validation involving GPT-3.5, the MAP4C SVM, the RNN, and finally the features SVM as negative control. For this purpose, we generated five data splits and conducted predictions anew.

**Fig. 1:**
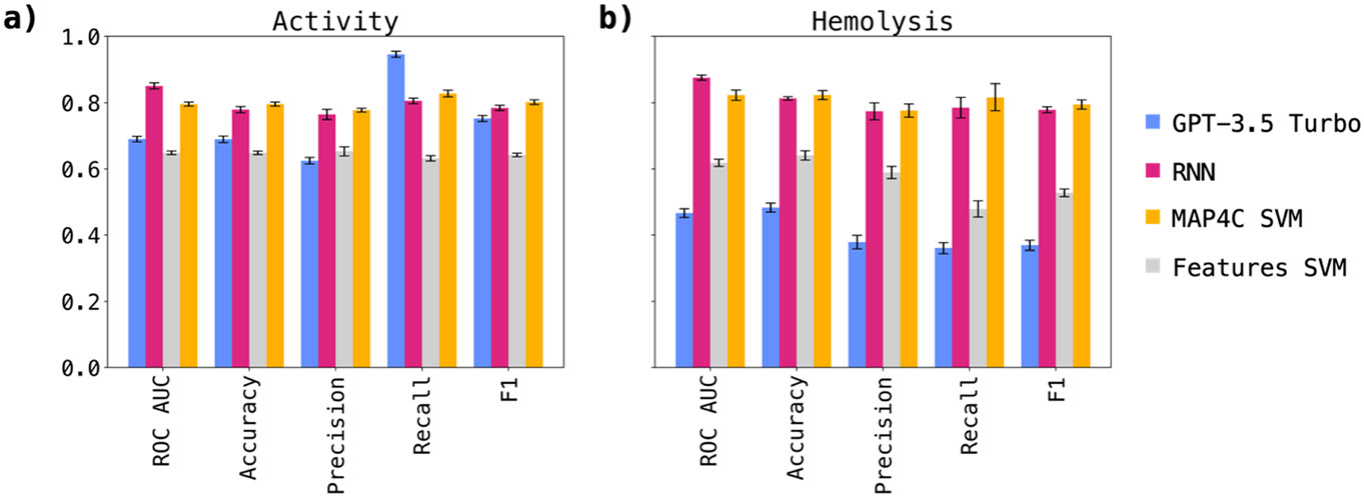
Results of the 5-fold cross-validation study aimed at validating MAP4C SVM, features SVM, RNN, and GPT-3.5 turbo performance for a) antimicrobial activity and b) hemolysis predictions. The mean performance across the 5 cross-validations for each metric is shown as a bar, the standard deviation is displayed with an error bar. The results confirmed earlier observations but showed notably higher performances for the RNN compared to the one-shot screening experiment. Both the RNN and MAP4C SVM demonstrated comparable performances.

The results, depicted in Fig. 1a for antimicrobial activity prediction and Fig. 1b for hemolysis prediction, confirmed our earlier observations (performances in Table S2†). Notably, the RNN performances were higher than those observed in the screening experiment, and were clearly above those of GTP-3.5. Furthermore, both the RNN and MAP4C SVM demonstrated comparable performances, indicating the validity of both approaches in predicting antimicrobial activity and hemolysis. The finding that simpler machine learning architectures, like SVM, can rival the performance of more complex RNNs in predicting antimicrobial activity and hemolysis is particularly interesting. A comparison with models trained on similar datasets, which achieve similar performances as reported in this study, further reinforces the consistency of our findings.^19–21^

This raises questions about the importance of model architecture *versus* foundational elements such as data quality and feature engineering. It suggests that a balanced approach, prioritizing optimization of these foundational components, could prove more beneficial than focusing solely on model complexity.

### Data visualization

The high performance achieved by the SVM trained on the MAP4C fingerprint suggested that the nearest neighbor relationships in the MAP4C feature space could be sufficient to distinguish active from inactive and hemolytic from non-hemolytic peptide sequences. In our previous work, we observed that the MAP4 fingerprint^38^ correctly clustered natural products, taken from the COCONUT database,^39^ according to their organism of origin.^40,41^ In analogy to our previous work, we were curious to see whether a spatial separation of actives/inactives and hemolytic/non-hemolytic sequences can be obtained from encoding with MAP4C, the chiral version of MAP4, possibly explaining the good performance of the MAP4C SVM model. For this, we reduced the 2048-dimensional feature space of MAP4C to 2D using the dimensionality reduction method TMAP,^42^ and used the obtained visualization to display a set of molecular properties.

**Fig. 2:**
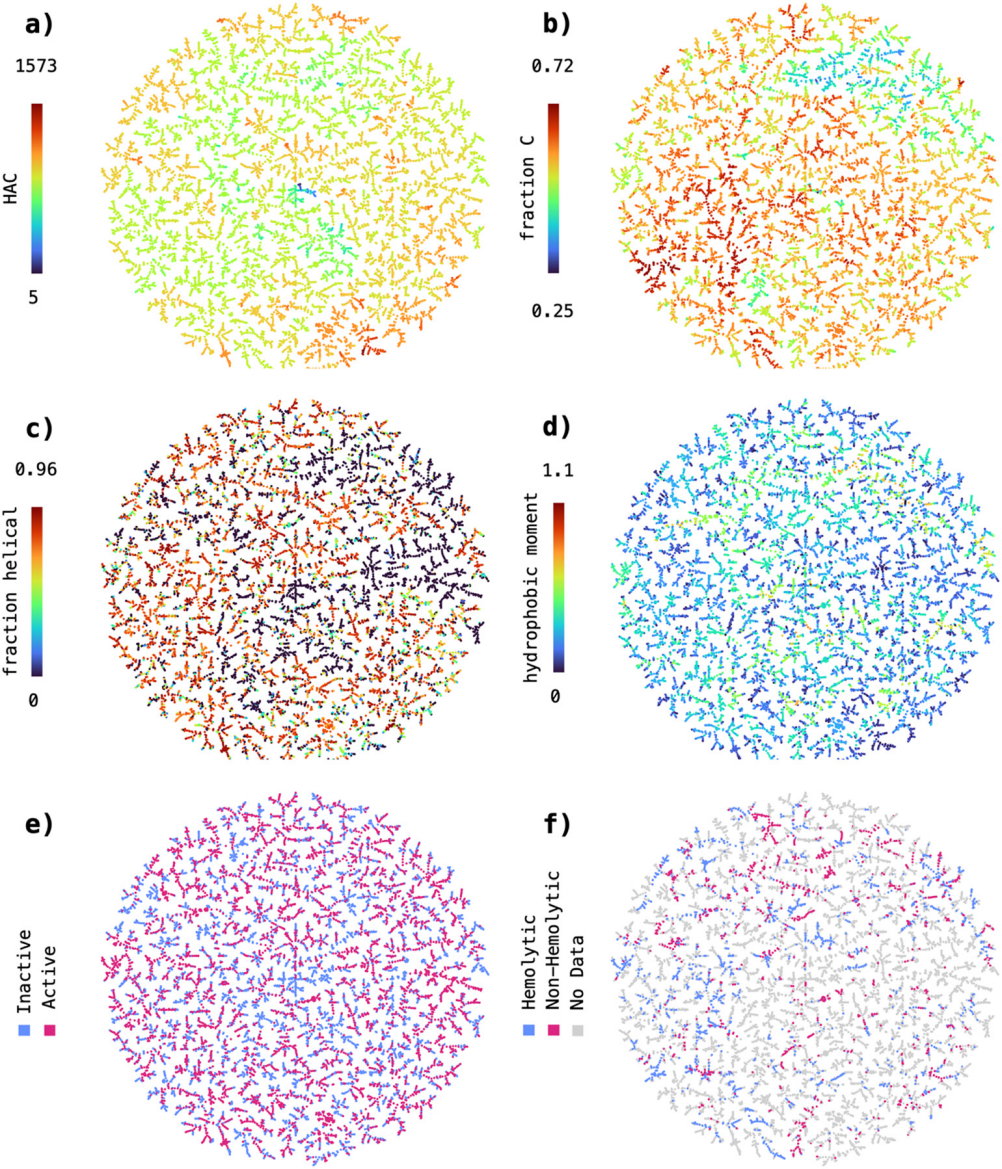
Chemical space covered by the 9548 peptide sequences with annotated antimicrobial activity extracted from the Database of Antimicrobial Activity and Structure of Peptides (DBAASP). The sequences are encoded using the MAP4C fingerprint and the resulting 2048-dimensional space reduced to 2D using TMAP. The sequences in the 2D TMAP were colored based on a) heavy atom count, b) fraction of carbon atoms, c) predicted fraction of helical residues, d) hydrophobic moment, e) annotated antimicrobial activity and f) annotated hemolysis.

First, we wanted to confirm that the TMAP visualization aligns with intuitive distributions of structural features relevant for peptides. For that, we colored the data points based on their heavy atom count (HAC), an indicator of molecular size, and fraction of carbon atoms (fraction C), a simple proxy for the hydrophobicity of a peptide sequence. The TMAP revealed visible clusters for both, HAC (Fig. 2a) and fraction C (Fig. 2b), indicating that the reduced MAP4C features can reliably represent simple molecular descriptors in the underlying chemical space.

Following this first observation, we wanted to test if we can detect clusters within TMAP visualizations of more complex physicochemical properties, such as the predicted fraction of helical residues (Fig. 2c) and the hydrophobic moment (Fig. 2d). In both cases, we could not detect large homogenous clusters as was the case for HAC and fraction C. However, the data formed a large number of small local clusters, indicating that the nearest neighbor relationships in the MAP4C feature space can possibly be used to distinguish sequences with high helicity/hydrophobicity opposed to sequences with low helicity/hydrophobicity.

Finally, we analysed the distribution of active *versus* inactive (Fig. 2e) and hemolytic *versus* non-hemolytic (Fig. 2f) sequences in the MAP4C chemical space. Similarly to the visualizations of predicted fraction of helical residues and hydrophobic moment, active and inactive or hemolytic and non-hemolytic sequences are spatially separated in a large number of small, local clusters. This finding is particularly interesting as it suggests that nearest neighbor relationships in the MAP4C feature space are sufficient to separate peptide sequences based on their antimicrobial activity and hemolysis. It further provides an explanation to the good performance obtained with the MAP4C SVM, which can leverage the nearest neighbor relationships stored in the MAP4C fingerprint feature space when provided with a custom Jaccard kernel function.

## Conclusion

In the present study we investigated the potential of LLMs as predictive tools for antimicrobial activity and hemolysis of peptide sequences. We assessed that fine-tuning GPT models in cloud is a relatively easy and fast process as access through the API eliminates the need to buy expensive hardware and requires little technical expertise. Duration of fine-tuning was short, and the associated costs were low (Table S3†). In contrast to cloud-based fine-tuning, local model training involves setting up and maintaining hardware, which can be costly and require technical expertise. While less complex models like RNNs and SVMs have lower hardware requirements, training larger models such as LLMs locally can pose challenges in terms of scalability, as one can rapidly face limitations in terms of hardware capacity and maintenance costs.

However, the lack of control over the training environment in cloud-based approaches raises concerns regarding reproducibility of scientific results. In the course of this study, we had originally fine-tuned GPT-3 models Ada, Babbage and Curie. These models performed slightly better than the reference models, even achieving good performances in low data regimes. Unfortunately, these models were later deprecated by OpenAI and their performance cannot be reproduced. When fine-tuning a newer iteration of GPT-3 (GPT-3.5 Turbo), we observed a significant decrease in performance for the same task. We attribute the drop in performance to the increasing optimization of LLMs for conversational interactions, which may negatively impact their effectiveness in out-of-scope predictive tasks. These findings highlight the potential risk of how not controlling one's own models can compromise the reproducibility and reliability of scientific results.

The aforementioned findings suggest a diminishing suitability of chat oriented LLMs for classification tasks over time, a function beyond their intended design. This observation specifically applies to LLMs tailored for conversational or human interaction purposes, rather than specialized LLMs trained on domain-specific data. Unfortunately, the latter do not provide the ease of access and usability that GPT models do. Consequently, we expect that LLMs will increasingly be employed in human interaction settings, facilitating the integration of various chemical tools through natural language interfaces as is being pioneered by Bran^31^ and Boiko *et al.*^32^

Finally, we could demonstrate in the present study that classical machine learning techniques, such as SVMs trained on MAP4C fingerprint encodings, can achieve state-of-the-art performance in the prediction of antimicrobial activity and hemolysis. This finding is especially interesting, as it showcases that good performance can be achieved by less complex models, putting the emphasis on data quality rather than model complexity.

## Code availability

The source codes and datasets used for this study are available at https://github.com/reymond-group/LLM_classifier.

## Author contributions

MO designed and realized the project and wrote the paper. JLR designed and supervised the project and wrote the paper. Both authors read and approved the final manuscript.

## Conflicts of interest

There is no conflict of interest to declare.

## Supplementary Material

MD-015-D4MD00159A-s001
