# Supplementary material for: Can large language models predict antimicrobial peptide activity and toxicity?
Source: RSC Med Chem. 2024 Apr 23;15(6):2030–6. doi: 10.1039/d4md00159a (PMC11187562; doi:10.1039/d4md00159a)
Supplement: MD-015-D4MD00159A-s001 [file MD-015-D4MD00159A-s001.pdf]

Electronic Supplementary Material (ESI) for RSC Medicinal Chemistry.
This journal is © The Royal Society of Chemistry 2024

Supplementary Information for:

Can Large Language Models Predict Antimicrobial Peptide Activity and Toxicity?

Markus Orsi,[a] and Jean-Louis Reymond[a]*

[a)] *Department of Chemistry, Biochemistry and Pharmaceutical Sciences, University of Bern, Freiestrasse 3, 3012 Bern, Switzerland*

e-mail: jean-louis.reymond@unibe.ch

Table of Contents

Table S1 2

Table S2 2

Table S3 3

Table S1. Performance metrics of all models tested on antimicrobial activity and hemolysis classification. The best value for each metric is highlighted in bold for activity and hemolysis separately. Results for reduced training sets are reported for 20% and 2% size of the original activity dataset and 10% of the original hemolysis set.

Model	ROC AUC	Accuracy	Precision	Recall	F1
GPT-3 Ada act.	0.84	0.78	0.78	0.78	0.78
GPT-3 Babbage act.	0.85	**0.79**	**0.79**	0.78	**0.79**
GPT-3 Curie act.	**0.86**	**0.79**	0.78	**0.81**	**0.79**
GPT-3 Ada 20% act.	0.75	0.69	0.7	0.67	0.68
GPT-3 Babbage 20% act.	0.76	0.69	0.7	0.69	0.68
GPT-3 Curie 20% act.	0.76	0.7	0.71	0.71	0.71
GPT-3 Ada 2% act.	0.66	0.6	0.6	0.63	0.61
GPT-3 Babbage 2% act.	0.66	0.62	0.6	0.73	0.66
GPT-3 Curie 2% act.	0.65	0.6	0.6	0.63	0.61
GPT-3 Ada hem.	**0.9**	0.82	0.8	**0.79**	0.79
GPT-3 Babbage hem.	0.87	0.8	0.76	0.76	0.76
GPT-3 Curie hem.	0.89	**0.84**	**0.82**	**0.79**	**0.8**
GPT-3 Ada 10% hem.	0.72	0.68	0.63	0.58	0.6
GPT-3 Babbage 10% hem.	0.72	0.7	0.65	0.6	0.62
GPT-3 Curie 10% hem.	0.73	0.68	0.63	0.59	0.61

Table S2. Mean and standard deviation of performance metrics of selected models tested on antimicrobial activity and hemolysis classification. The best value for each metric is highlighted in bold.

Model	ROC AUC	Accuracy	Precision	Recall	F1
Features SVM act.	0.65 ± 0.01	0.65 ± 0.01	0.65 ± 0.01	0.63 ± 0.01	0.64 ± 0.01
MAP4C SVM act.	0.8 ± 0.01	**0.8 ± 0.01**	**0.78 ± 0.01**	0.83 ± 0.01	**0.80 ± 0.01**
RNN act.	**0.85 ± 0.01**	0.78 ± 0.01	0.76 ± 0.02	0.81 ± 0.01	0.78 ± 0.01
GPT-3.5 Turbo act.	0.69 ± 0.01	0.69 ± 0.01	0.62 ± 0.01	**0.95 ± 0.01**	0.75 ± 0.01
Features SVM hem.	0.62 ± 0.01	0.64 ± 0.01	0.59 ± 0.02	0.48 ± 0.02	0.53 ± 0.01
MAP4C SVM hem.	0.82 ± 0.02	**0.82 ± 0.01**	**0.78 ± 0.02**	**0.82 ± 0.04**	**0.79 ± 0.01**
RNN hem.	**0.87 ± 0.01**	0.81 ± 0.01	0.77 ± 0.03	0.79 ± 0.03	0.78 ± 0.01
GPT-3.5 Turbo hem.	0.47 ± 0.01	0.48 ± 0.01	0.38 ± 0.02	0.36 ± 0.02	0.37 ± 0.02

Table S3. Training times and costs of GPT models on the full training sets.

**Model**	**Time (h)**	**Costs ($)**
GPT-3 Ada Activity	01:05:04	$0.39
GPT-3 Babbage Activity	01:09:38	$0.59
GPT-3 Curie Activity	01:15:05	$2.93
GPT-3.5 Turbo Activity	00:53:24	$7.00
GPT-3 Ada Hemolysis	00:55:37	$0.09
GPT-3 Babbage Hemolysis	00:57:19	$0.13
GPT-3 Curie Hemolysis	01:08:09	$0.67
GPT-3.5 Turbo Hemolysis	00:55:58	$1.66
